# Letter Regarding “Improvement in Nasal Symptoms of Chronic Rhinitis after Cryoablation of the Posterior Nasal Nerve”: Toward a Unified Airway Approach

**DOI:** 10.1002/oto2.153

**Published:** 2024-06-01

**Authors:** Diego M. Conti, Eduardo J. Correa

**Affiliations:** ^1^ The European Forum for Research and Education in Allergy and Airway Diseases Scientific Expert Team Members Brussels Belgium; ^2^ Escuela de Doctorado UAM, Centro de Estudios de Posgrado Universidad Autónoma de Madrid, Ciudad Universitaria de Cantoblanco Madrid Spain; ^3^ Otolaryngology Department Hospital Comarcal de la Línea de La Concepción Cádiz Spain

Dear Editor,

Our workgroup has read with interest the article *Improvement in Nasal Symptoms of Chronic Rhinitis after Cryoablation of the Posterior Nasal Nerve* by Rosi‐Schumacher et al^1^ and have concerns about the methodology employed and the results obtained.

Although brief, reference is made to the impact on patients' quality of life. However, only those aspects related to nasal obstruction or nasal discharge are mentioned, not those leading to associated comorbidities. This was not addressed or taken into account, even though patients reported such comorbidities in the questionnaire used, and no distinction was made between patients with rhinitis alone and those with associated conditions such as asthma or chronic rhinosinusitis. In this way, the concept of a unified airway is neglected or omitted. Our recommendation is to investigate associated comorbidities and reconsider the primary diagnosis before increasing the complexity of therapy.^2^, ^3^

The use of the above procedure is justified on the basis of nonresponse to standard treatments and the authors state the economic burden of allergic rhinitis, but no reference is made to the cost of the procedure in the introduction. It should also be noted that there is no mention of whether this is a one‐stage procedure or whether further interventions may be required^4^. Furthermore, it should not be underestimated that cryoablation is an invasive procedure and, although mentioned, it is not without side effects and contraindications.

Given that cryoablation is a palliative, symptomatic rather than curative treatment for allergic rhinitis, we note that patients were not encouraged to continue medical treatment and follow‐up after procedure, as prescribed before. Perhaps it would be in the interest of both the patient and the health care system to anticipate this, assess the reasons for therapeutic failure and add therapies that provide long‐term benefit, such as allergen‐specific immunotherapy.^5^

Finally, although mentioned in the text, this study has the major limitation of a short follow‐up, with a mean of 51.8 days, which seems rather short for a chronic disease. This is critical as the conclusions are based on the results of cryoablation, which have not been measured in the long term. Perhaps a new intervention in a prospective study would address this weakness.

In summary, this group believes that consideration of rhinitis as a co‐morbidity or associated co‐morbidities would have had a decisive influence on sample selection and the decision to advance in therapeutic complexity. The concept of a unified airway is compelling, but has been omitted.

## Author Contributions

**Diego M. Conti**, has made substantial contributions to the conception and design of the work, has contributed to the interpretation of data, has drafted the work, and have revised it substantially and also approved the version submitted and has agreed both to take personal responsibility for their own contributions and to ensure that questions concerning the accuracy or integrity of any part of the work are appropriately investigated, resolved and the resolution documented in the literature; **Eduardo J. Correa**, has made substantial contributions to the conception and design of the work, has contributed to the interpretation of data, has drafted the work and have revised it substantially and also approved the version submitted and has agreed both to take personal responsibility for their own contributions and to ensure that questions concerning the accuracy or integrity of any part of the work are appropriately investigated, resolved and the resolution documented in the literature.

## Disclosures

### Competing interests

Diego M. Conti, serves as academic director at the European Forum for Research and Education in Allergy and Airway Diseases (EUFOREA) and guest associate editor at Frontiers in Allergy, rhinology section. Eduardo J. Correa, no conflicts of interest related to this initiative.

### Funding source

This initiative has not received any funding or financial support.
